# 360°VR: Application for exercise and sport science education

**DOI:** 10.3389/fspor.2023.977075

**Published:** 2023-03-20

**Authors:** Aden Kittel, Michael Spittle, Paul Larkin, Sharna Spittle

**Affiliations:** ^1^ Institute for Health & Sport, Victoria University, Melbourne, VIC, Australia; ^2^ Maribyrnong Sports Academy, Melbourne, VIC, Australia; ^3^ College of Sport and Exercise Science, Victoria University, Melbourne, VIC, Australia

**Keywords:** immersive 360° video, virtual reality, higher educaction, 360 VR, sport science, career development, soft skills

## 1. Introduction

Soft skills are integral for early career success for higher education graduates (1). An example of an industry where these skills are vital is Exercise and Sport Science (ESS) (2), where over 2,000 students each year from 30+ Universities graduate (3). Developing these soft skills supports graduates to extend beyond technical skills and be productive and competent in a dynamic workplace (4). However, technical skills such as the development of knowledge have received a stronger emphasis in the ESS curriculum compared to transferable skills such as communication and developing positive relationships (2). The aim of this opinion paper is to propose 360°VR as an authentic learning tool for developing vital soft skills in ESS education, which are typically underdeveloped. 360°VR will be critically assessed through a SWOT analysis (strengths, weaknesses, opportunities, threats), with several examples presented and discussed.

## 2. Exercise and sport science overview

ESS is a multidisciplinary field that has developed significantly in recent decades (5). The definition of ESS varies globally, with the British Association of Sport and Exercise Sciences (BASES) defining ESS as “the application of scientific principles to sport and exercise, achieved through one of the following three branches of science (biomechanics, physiology, psychology), or through interdisciplinary approaches” (6). However, in Australia, the accrediting body, Exercise and Sports Science (ESSA) defines a Sport Scientist as a professional who “provides expert advice and support to athletes and coaches to help them understand and enhance sports performance; adopting evidence-based, quality-assured practice to evaluate and develop effective strategies or interventions in training and/or competition” (7). ESSA (7) also describes ESS tertiary education as following a holistic approach including foundational knowledge in biomechanics, physiology and psychology, but also anthropometry, training methodology, motor learning and learning. Given the multidisciplinary nature ESS, there are a variety of career paths for tertiary graduate students, with the most common including exercise physiologist, strength and conditioning coach, sport scientist, high performance manager, sport physiologist, and academic (8).

## 3. Skills and attributes for ESS

There are a number of skills to practice effectively in ESS-related careers. Bruce et al. (2) analysed the perceived importance of key skills for ESS roles, as viewed by those working in the sport science industry from an academic and applied perspective. The most important technical skills included contemporary and sport-specific research and best practice knowledge; practicing in an inclusive/non-discriminatory manner; being able to analyse the demands of the sport/athlete capabilities; and ability to analyse data's validity and reliability. Although these technical skills are important for practitioners, “soft skills” (e.g., transferable, interpersonal skills) may be important in supporting these technical skills, by enabling effective knowledge translation through strong communication and relationships (9). The perceived importance of soft skills have grown over the last decade, and include interpersonal, intellectual and practical skills, allowing individuals to behave positively and adapt to professional challenges (10). Examples of important transferable skills for ESS graduates include written/oral communication, identifying and using appropriate communication techniques, creating positive professional relationships with stakeholders, and ability to adapt to contextual/role demands through adaptive thinking (2). These transferable (i.e., “soft”) skills were consistently rated to be more important by those in applied settings than academic (2). Soft skills are necessary in the most common ESS career paths, which are all service-related (8). In strength and conditioning, simulated practice environments such as work-integrated learning were the best pedagogical approaches to develop skills (11).

Developing these soft skills supports graduates to extend beyond technical skills and be productive and competent in a dynamic workplace (4). While it may be more difficult to develop soft skills in the classroom than technical skills, work-integrated learning (i.e., placement) is an important opportunity for ESS students to develop key soft skills (12). By developing communication and interpersonal skills in placements, this facilitates a smoother transition to the workplace (12, 13). There are only a finite amount of hours students can engage in placement activities in non-paid capacity, to avoid exploitation of students seeking to gain experience and not take away from the curriculum time required to develop technical skills/knowledge (12). Therefore, implementing more innovative and authentic learning activities in the classroom may be an approach to develop these skills.

## 4. Pedagogical framework: authentic learning

The pedagogical stance adopted for this paper is authentic learning, which can increase the employability of students by developing skills necessary for the workplace (14). Authentic learning approaches refer to role-playing and problem-based exercises that focus on real-world, complex problems and their solutions in multidisciplinary learning environments (15). Given the multidisciplinary nature of the ESS field of study (3, 5), authentic learning appears to be appropriate for developing key skills. An example of an innovative authentic learning approach is mixed reality technologies, such as 360° Virtual Reality (360°VR) as recommended by Stanley (16). This technology offers the possibility to allow students to learn complex problems in an appealing and stimulating manner, while being more engaged and motivated in their study (16, 17) Novel curricula activities and technologies such as 360°VR could be developed to provide opportunities to develop key workplace soft skills within an authentic learning environment.

## 5. 360°VR as an authentic learning tool

360°VR (also defined as immersive video) captures real-world video using a 360° camera. This technology can be differentiated from Virtual Reality (VR) that uses virtual/animated environments similar to a video game. Kittel et al. (18) provide a succinct differentiation between these two technologies. While 360°VR can be viewed on a screen/monitor with a mouse to move the video, 360°VR is most commonly presented using a head mounted display (HMD) allowing individuals to scan the environment, with visual information matched to head movements like real life. As such, 360°VR has been labelled an appropriate “middle ground” between screen-based videos and VR, given it allows greater interaction with scanning the environment through head movements (19). 360°VR has been investigated as an authentic educational tool in teacher education (20, 21); environmental conservation (22); medical and surgical training (23); safety management (24); and inter-professional communication in healthcare (25). The use of this technology has grown in recent years (see Pirker and Dengel (26) for a review). Despite the increased use in this technology, coupled with the identified need for more authentic learning tools in ESS education, 360°VR has not yet been investigated in this area.

## 6. SWOT analysis of 360°vr in ESS education

A SWOT analysis is an effective strategic planning tool to analyse the strengths, weaknesses, opportunities and threats of a new instrument/process (27, 28). While SWOT analyses have investigated 360°VR in sport (18) and teacher education (20), no such SWOT analysis has been conducted in ESS tertiary education. This is pertinent given the expanding nature of the field of ESS (5) and rise of 360°VR technology (26).

### 6.1. Strengths

Authentic learning provides a more engaging learning environment, with 360°VR an appropriate technology for this pedagogical approach (16, 17). This is supported in other domains such as sports training, where Kittel et al. (29) report 360°VR to be a more enjoyable and relevant tool than screen-based video. 360°VR provides a technologically advanced tool, which can be more engaging for current, tech-savvy generations (25). Within an immersive 360° environment, the learner has less of a passive perspective than when viewing a monitor/screen, as they can choose what to watch and engage (20). For example, research in teacher education has indicated that a short intervention of 360°VR (3 × 2 h sessions) can lead to improvement in inter-professional vision, where learners develop the ability to identify key elements in their working environment (30).

In addition, a key soft skill for early career success is the ability to manage stress (1), and Theelen et al. (21) also reported that 360°VR exposure prior to work experience led to a decrease in anxiety, and subsequent increased self-efficacy. Work-integrated learning is a vital opportunity for students to apply theoretical knowledge gained in higher education to a real-world setting (2, 31), yet there are only a finite amount of hours and opportunities students can participate in work-integrated learning. Ranieri et al. (32) highlight 360°VR can lead to greater transfer of theoretical knowledge through practical scenarios, therefore providing a valuable supplementary tool to work-integrated learning.

### 6.2. Weaknesses

360°VR can increase cognitive load (26), however Kittel et al. (29) reported no difference in concentration and effort for 360VR compared to screen-based video. Bartlett and Drust (9) highlight the importance of applying knowledge and developing relationships in ESS. 360°VR may not effectively develop these attributes as learners cannot directly interact with the immersive environment because of the view-only nature of this technology (20). Further, viewing 360°VR in a HMD may lead to feelings of discomfort (30). To overcome this, it is possible to view 360°VR on a monitor/screen, whereby learners can use a mouse to view the immersive space. However, this may limit the fidelity of the environment (i.e., extent to which the participant feels it is real) (33), as 360°VR viewed through a HMD affords stronger psychological fidelity than screen-based approaches (34).

### 6.3. Opportunities

**Figure 1:**
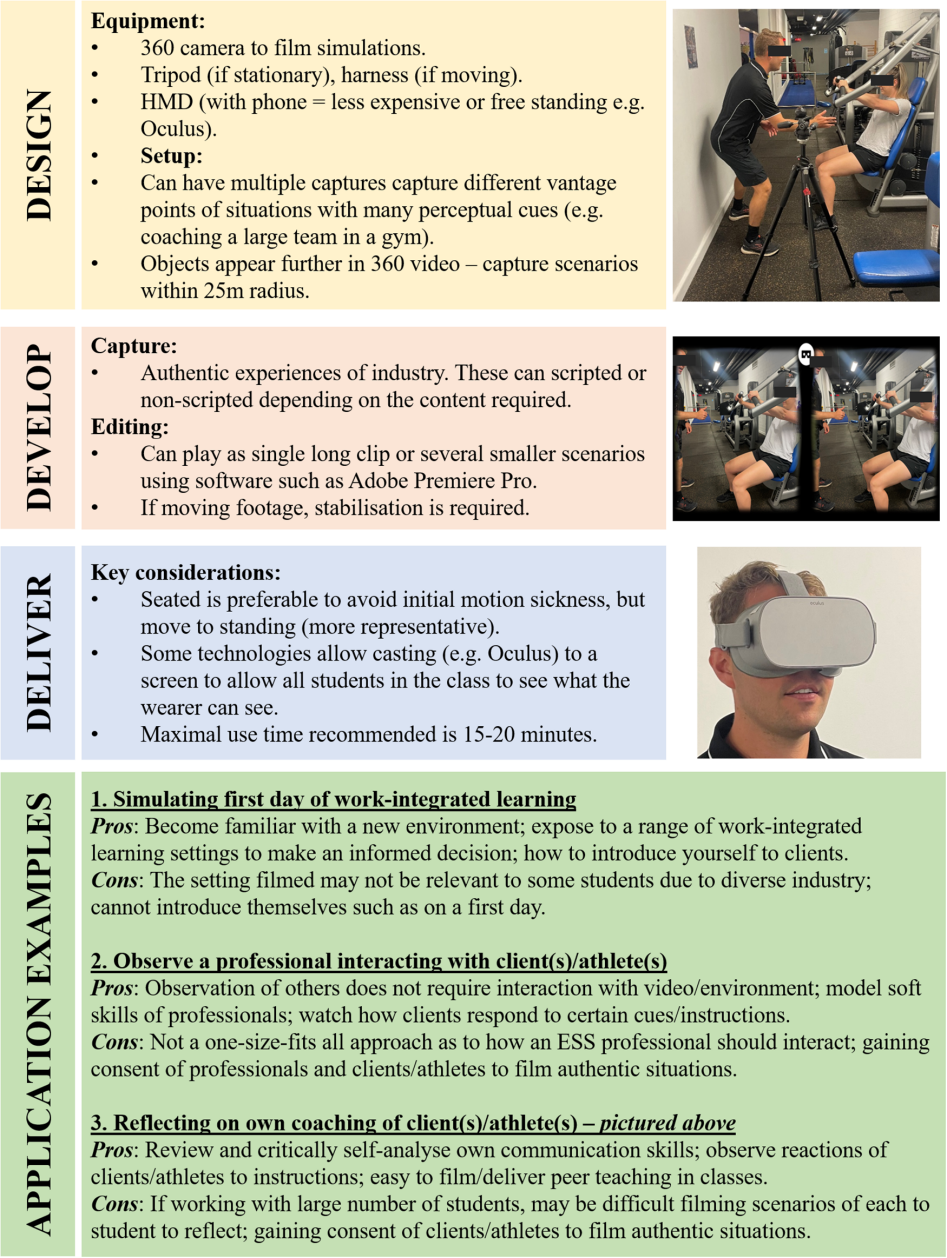
An overview of how to design, develop and deliver 360°VR in ESS higher education, with examples of applications.

There a range of applications possible for 360°VR in ESS higher education. Figure 1 presents a short guide for academics and professionals on how to design, develop, and deliver 360°VR in ESS higher education. Additionally, the pros and cons of three example applications for this technology are presented, which could be implemented and assessed in future research and practice. These opportunities discussed align to previous literature in this section. Although the examples highlighted focus on ESS education, these could be addressed in similar industries such as sport management or physiotherapy.

Prior to undertaking work-integrated learning experiences, Carson et al. (11) recommend implementing authentic simulated practice environments to allow gradual progression for students, such as in teacher education (30). 360°VR provides an opportunity to facilitate this gradual progression from the classroom to real-world settings as an authentic learning simulation (16). Given the multidisciplinary nature of ESS, students can have a limited understanding of broader opportunities in the industry before entering the workforce (35). 360°VR can introduce students to these opportunities, or reignite motivations behind choosing their degree (3) in an immersive experience. A potential approach to provide this introduction or motivation is conducting virtual field trips (16, 36).

Figure 1 highlights that 360°VR can be casted to a screen when delivered using certain HMDs (e.g., Oculus). By purchasing more expensive technology that can cast to a screen, this will allow more students to view the same video. Although 360°VR has limited environmental interaction, this technology provides an opportunity to observe others’ interpersonal behaviour strategies, as in teacher education (30). This affords an opportunity for students in ESS to observe and reflect on their own or others' interpersonal skills (20), which may be important, given the importance of these skills (2). Work-integrated learning in ESS allows students to reflect on their own practice (37). 360°VR can provide a valuable supplementary tool, whereby students film themselves and/or their peers to promote greater reflection. James et al. (38) discuss how ESS students entering the strength and conditioning industry require more practical coaching experience, and industry professionals may benefit from more reflective practice. As Figure 1 identifies the possibilities for reflective and observational practice, 360°VR has an opportunity to be embedded as a professional development tool for students and professionals in this industry.

### 6.4. Threats

360°VR can induce motion sickness/discomfort (39), with females more prone to experience motion sickness in VR environments (40). It is vital to promote opportunities for females in sports-related industries, as this has historically been male-dominated (41). More research needs to be conducted to explore the factors to prevent motion sickness in females using VR, to promote this as an educational tool. In comparison to more traditional technologies such as screen-based video, 360°VR is more expensive and difficult to capture (18, 20). Providing all class members access to the video can be a limitation. To overcome this, it is recommended to purchase less-expensive HMDs where the students can input their smartphones, allowing an entire class to view the 360°VR. In addition, producers of 360°VR must be aware of ethical considerations when filming authentic situations. Threats such as these are identified as cons of application examples presented in Figure 1. While 360°VR is a more engaging education tool in some domains, some students may not use this technology due to financial and portability concerns (42).

## 7. Conclusion

To summarise, authentic learning environments are key in the development of ESS educational programs. By providing authentic experiences, students can develop key soft skills perceived as vital for future employment and career development, yet may not be emphasised in the ESS curriculum. 360°VR presents a novel authentic learning tool to develop skills and attributes for ESS students in a more engaging manner. While practitioners, educators, and researchers should be aware of the limitations of this technology, more research is required to understand its' application in ESS higher education. It is anticipated that the implications of the current opinion will stimulate the use of 360°VR in similar areas (e.g., sport management, physiotherapy), or areas also requiring stronger emphasis on soft skill development in higher education.
